# The Role of Personal Experience and Prior Beliefs in Shaping Climate Change Perceptions: A Narrative Review

**DOI:** 10.3389/fpsyg.2021.669911

**Published:** 2021-07-02

**Authors:** Kate Sambrook, Emmanouil Konstantinidis, Sally Russell, Yasmina Okan

**Affiliations:** ^1^School of Earth and Environment, Sustainability Research Institute, University of Leeds, Leeds, United Kingdom; ^2^Department of Psychology, University of Warwick, Coventry, United Kingdom; ^3^Centre for Decision Research, Leeds University Business School, University of Leeds, Leeds, United Kingdom

**Keywords:** climate change, extreme weather, personal experience, prior beliefs, climate change beliefs

## Abstract

Global climate change is increasing the frequency and intensity of extreme weather events such as heatwaves, droughts, and flooding. This is the primary way many individuals experience climate change, which has led researchers to investigate the influence of personal experience on climate change concern and action. However, existing evidence is still limited and in some cases contradictory. At the same time, behavioral decision research has highlighted the importance of pre-existing values and beliefs in shaping how individuals experience changes in environmental conditions. This is in line with theories of motivated reasoning, which suggest that people interpret and process information in a biased manner to maintain their prior beliefs. Yet, the evidence for directional motivated reasoning in the context of climate change beliefs has recently been questioned. In the current paper, we critically review the literature on the interrelationships between personal experience of local weather anomalies, extreme weather events and climate change beliefs. Overall, our review shows that there is some evidence that local warming can generate climate change concern, but the capacity for personal experience to promote action may rely upon the experience first being attributed to climate change. Rare extreme weather events will likely have limited impact on judgments and decisions unless they have occurred recently. However, even recent events may have limited impact among individuals who hold strong pre-existing beliefs rejecting the reality of climate change. We identify limitations of existing research and suggest directions for future work.

## Introduction

Climate change is a global challenge that has already had a detrimental impact on the environment and human health, leading to increases in the magnitude and frequency of extreme weather events such as heatwaves, droughts and flooding (Seneviratne et al., 2012). Despite compelling evidence for rapid climate change and the urgency of the issue (IPCC, 2018), some individuals remain skeptical about the risk and reality of climate change (Whitmarsh, 2011). A key factor that can shape perceptions of climate change is people's personal experience of extreme weather events and/or local weather anomalies such as temperature fluctuations (i.e., deviations from the normal seasonal temperature) (Spence et al., 2011; Zaval et al., 2014; Demski et al., 2017). Such experiences provide an opportunity for individuals to witness the otherwise abstract effects of climate change and as such make the risk more tangible and familiar (Lorenzoni and Pidgeon, 2006; Weber, 2010; Spence et al., 2011; Smith and Joffe, 2013; Reser et al., 2014). Specifically, such experiences can reduce perceived psychological distance of climate change from the self on different dimensions, including temporally, socially and geographically (McDonald et al., 2015). Furthermore, experiences with extreme weather events may also provoke negative affective responses, which can increase perceived risk (Keller et al., 2006), increase environmental concern and promote action (Demski et al., 2017; Bergquist et al., 2019).

However, personal experiences of climate change impacts may not always increase concern or motivate action. The literature on motivated reasoning suggests that people often process new information in a biased way to generate their favored conclusion and maintain their prior beliefs (Kunda, 1990; Dawson et al., 2002; Myers et al., 2013). As such, individuals' prior beliefs about climate change may influence how they interpret fluctuations in local weather conditions. This can substantially reduce the likelihood of skeptical individuals acknowledging the reality and consequences of climate change, which in turn could lower their willingness to adopt adaption (e.g., paying for flood damage insurance) and/or mitigation actions (e.g., deciding to travel by train rather than flying; Lorenzoni and Pidgeon, 2006; Weber, 2006).

In this mini review, we provide an overview of existing literature on the interrelationships between personal experience of local weather anomalies, extreme weather events and climate change beliefs. We first review studies examining whether personal experiences shape beliefs and actions, and outline moderating factors, considering relevant literature in behavioral decision research. Next, we review work examining the influence of pre-existing climate change beliefs on interpretations of weather-related experiences, in connection to work on motivated reasoning. Finally, we identify limitations of existing research and suggest directions for future research. An overview of the basic characteristics of all studies reviewed and main results for each study can be found in the online Supplementary Materials.

## Does Personal Experience Shape Climate Change Beliefs and Action?

There is evidence to suggest that subjective (self-reported) experiences of local weather anomalies could increase belief in and concern for climate change (Krosnick et al., 2006; Howe and Leiserowitz, 2013; Zaval et al., 2014; Howe, 2018). For example, survey respondents in Australia and the United States (U.S.) who reported warmer-than-usual temperatures on the day of study expressed greater belief in and concern about climate change (Li et al., 2011). However, these studies are limited by the cross-sectional and self-reporting nature of the data and hence a causal relationship between personal experience and climate change beliefs cannot be established.

Studies that have measured the effect of observed temperature fluctuations on climate change beliefs provide evidence that abnormally warm temperatures in the short term (Joireman et al., 2010; Egan and Mullin, 2012; Hamilton and Stampone, 2013) and the long term (Deryugina, 2013; Shao et al., 2014, 2016; Shao, 2017) are important predictors of climate change beliefs and risk perceptions. For example, three 10-year studies in the U.S. reported a positive relationship between increasing summer temperatures and belief in the immediate impacts and severity of climate change (Shao et al., 2014, 2016). Other studies, however, did not find clear associations between short and long-term local temperature fluctuations and climate change concern (Li et al., 2011). Shao et al. (2016) and Deryugina (2013) argued that this may be due to differences in measurements of short-term temperature (i.e., monthly rather than daily data) and survey questions used to assess climate change beliefs. Alternatively, Brody et al. (2008) indicated a possible misunderstanding of the risks presented by long-term temperature change on individual well-being.

A smaller number of studies have examined specifically whether experiences of extreme weather events influence perceptions of climate change. For example, early research found no differences in climate change concern between flood and non-flood victims in the United Kingdom (U.K.) (Whitmarsh, 2008), with respondents reflecting a view that flooding was a separate issue from climate change and was instead caused by local changes such as road resurfacing.

Contrasting with these initial findings, more recent studies have found that personal experience with severe storms and associated floods (Spence et al., 2011; Taylor et al., 2014; Lujala et al., 2015), hurricanes (Bergquist et al., 2019), heatwaves (Dai et al., 2015), and droughts (Carmichael and Brulle, 2017) can influence climate change beliefs and concern, at least temporarily. For example, Spence et al. (2011) found that respondents in the U.K. who reported direct flooding experience expressed a higher level of concern about climate change impacts, compared to non-flood victims. Similarly, Demski et al. (2017) documented an increase in concern about climate change, as well as heightened personal salience of climate change issues and negative emotional responses following flooding experiences. Beyond flooding experiences, Dai et al. (2015) recorded participants' experiences of several extreme weather events (e.g., heavy rainfall, heatwaves, droughts, and avalanches) in five Chinese cities and found correlations between perceived experiences (particularly heatwaves) and climate change beliefs.

There is also some evidence that personal experience of extreme weather events may motivate individuals to act on climate change (Spence et al., 2011; Broomell et al., 2017). For example, Demski et al. (2017) showed that higher levels of personal and local threat from climate change following a flooding experience can prompt actions such as changing to a green energy supplier, as well as support for climate-related policy. Similarly, a study in Vietnam illustrated that flooding experience was strongly associated with intentions to implement adaption actions (Ngo et al., 2020). Other studies, however, suggest that experience of extreme weather events is associated with perceived threat from climate change, but not with willingness to take action (Whitmarsh, 2008; Brulle et al., 2012; Carlton et al., 2016). For example, a recent survey found that self-reported flooding experiences significantly predicted perceived threat from climate change but did not influence mitigation intentions (Ogunbode et al., 2019).

## Factors Moderating the Impact of Personal Experience on Climate Change Beliefs and Action

Overall, the empirical evidence on the relationship between personal experience of extreme weather events and climate change concern and action remains mixed. The contrasting findings may partly reflect that these experiences only generate climate change concern and action under particular circumstances that can affect memory strength for events and their impact on decisions, namely when events are: (1) relatively recent (Konisky et al., 2016); (2) linked with significant personal and/or financial damages (Lujala et al., 2015; Sisco et al., 2017); (3) experienced as abnormalities in temperature (Sisco et al., 2017); and (4) attributed to climate change (Ogunbode et al., 2019). We discuss these moderating factors in more detail below.

Firstly, more recent extreme weather events may have a significant impact on climate change concern and action, due to the ease with which they are recalled (Keller et al., 2006). In recent years, research on risky choice has investigated how personal experience affects judgment and decision making under risk. One aspect of this line of research has focused on rare (and extreme) events; This is particularly relevant in the context of climate change as most (extreme) weather events (e.g., heatwaves, flooding) have a small probability of occurrence. When making decisions based on experience such rare events are generally *underweighted* (i.e., their impact on making choices/taking action is smaller than their objective probability suggests). This is because their probability of having recently occurred is (on average) small. However, when they occur, their impact on future decisions can be larger than what is warranted by their objective probability, suggesting strong sequential dependencies and recency effects (Hertwig et al., 2004; Weber, 2010). This implies that personal experience of extreme (and rare) weather events can cause unstable effects on climate change beliefs, and that the specific nature of personal experience is a key determinant. For example, recency effects can be amplified if the event was linked with personal and financial damage (Sisco et al., 2017). Similarly, a study in Norway showed that respondents who had suffered damage from a climate-related event such as flooding or a landslide were 40% more likely to be concerned about climate change (Lujala et al., 2015). However, as it has already been mentioned [see Ogunbode et al. (2019)], experiencing extreme weather events may not necessarily lead to taking action, while at the same time may increase concern about climate change. This is similar to what Barron and Yechiam (2009) observed in a sequential risky choice task: while people's choices showed reduced sensitivity to rare events (i.e., underweighting), their probability judgments/estimations suggested overestimating the occurrence of such events. Recent research has attempted to unpack aspects of this paradoxical finding, for example whether people's choices and judgments are susceptible to biases arising from misinterpreting sequential patterns (similar to gambler's fallacy; Plonsky et al., 2015; Szollosi et al., 2019; see also Ashby et al. (2017)].

Further, as highlighted by Li et al. (2011) heightened temperatures on the day/days leading up to a study are associated with increased belief in and concern about climate change (Joireman et al., 2010; Egan and Mullin, 2012; Brooks et al., 2014). For example, in one study, belief that climate change is happening was predicted by temperature anomalies (i.e., unseasonable warm and/or cool temperatures) on the interview day and the previous day (Hamilton and Stampone, 2013). However, individuals must attribute these experiences to climate change to not only increase concern for climate change, but also to encourage action (Reser and National Climate Change Adaptation Research Facility, 2011; Akerlof et al., 2013; Myers et al., 2013). That is, when an individual experiences the impacts of climate change (i.e., extreme weather), such experience may not affect their concern or willingness to take action unless they first make a causal connection between the experience and climate change (Weber, 2010; Helgeson et al., 2012). To illustrate, individuals affected by severe flooding in the U.K. in 2013/2014, reported a greater perceived threat from climate change, but only those who attributed the event to climate change supported mitigation actions (Ogunbode et al., 2019). This points to the important role of pre-existing beliefs in shaping people's interpretations and attributions of different weather events.

## Do Prior Beliefs Shape an Individual's Perception of Climate Change Impacts?

Given that a single extreme weather event does not necessarily reflect long-term climate change trends, individuals may rely on their pre-existing beliefs to interpret extreme weather events in terms of climate change. This implies that individuals who already believe in climate change may interpret their experiences as a confirmation of the impacts of climate change. Instead, those who are more skeptical may be less likely to attribute their experiences to climate change (Howe and Leiserowitz, 2013). Such belief-driven interpretations may reduce the likelihood of some individuals becoming concerned about climate change (Howe and Leiserowitz, 2013; Broomell et al., 2017).

The influence of prior beliefs on climate change perceptions can be explained through a process of directional motivated reasoning. In broad terms, the concept describes how individuals tend to interpret and process information in a biased way that confirms their prior beliefs (Druckman, 2015). Such processing can affect the interpretation of new climate change information and experiences and may motivate individuals to reach their preferred conclusion regardless of accuracy or credibility (Druckman, 2015). The importance of directional motivated reasoning in shaping interpretations of extreme weather events has been documented frequently in recent years (Hart and Nisbet, 2012; Dietz, 2013; Druckman, 2015; Kahan and Corbin, 2016). For example, a national U.S. survey found that respondents who lived in places that were affected by a heatwave in 2010 but were “doubtful” or “dismissive” about climate change were 27% less likely to report experiencing a warmer-than-normal summer (Howe and Leiserowitz, 2013). These findings suggest that motivated reasoning may bias recall of local climate, particularly among those who do not believe in the existence of climate change. Other studies have also indicated that prior beliefs and cultural orientations can bias people's recollections of local weather experiences (Goebbert et al., 2012; Shao, 2016). To illustrate, Shao (2016) found that individuals who believed that climate change is having impacts now were more likely to perceive a strange pattern of weather in the past. Meanwhile, results from a longitudinal survey by Myers et al. (2013) showed that highly engaged individuals (i.e., those who were either strongly convinced of the reality of climate change or strongly rejected it) were more likely than less engaged ones to interpret their personal experiences in a way that strengthened their pre-existing beliefs. It should be noted however, that this and other studies examining the effect of prior beliefs on reported experience of extreme weather events are primarily limited to regions within North America, which has, in recent decades, become deeply divided on the issue of climate change (Carmichael and Brulle, 2017).

Another useful illustration of the role of prior beliefs is research that examines associations between political indicators (i.e., ideology and party identification) and climate change perception (McCright et al., 2014; Hamilton et al., 2015; Zanocco et al., 2018; Marlon et al., 2019). For example, between 2010 and 2014, Palm et al. (2017) showed that Democrats were convinced climate change was occurring and demanded action, whereas Republicans remained skeptical about climate change. Importantly, such views were generally strengthened over time in both groups, particularly among those engaged with the news and public affairs. This finding is consistent with a process of politically motivated reasoning whereby individuals process information in a way that aligns with their party ideology. Similarly, in a survey across four Gulf Coast states, Shao and Goidel (2016) found that compared with Republicans, Democrats were more likely to believe in the existence of climate change and demonstrated greater concern for future consequences. In addition, Democrats were more likely to notice changes in local weather conditions, including an increase in the frequency and intensity of hurricanes, droughts and flooding.

There is also evidence that motivated reasoning may not affect the recall of all past experiences equally. Memories of abnormal local temperatures are more likely to be shaped by climate change beliefs than other weather types (precipitation), due to the natural link between climate change (global warming) and temperature (Leiserowitz, 2006). Evidence from two national surveys in Norway and the U.S. support this view and find that perceptions of seasonal temperature were shaped by climate change beliefs, political ideology and cultural biases (Goebbert et al., 2012; Howe, 2018). The relationship was substantially weaker for local precipitation, floods and droughts.

Finally, it should also be noted that the evidence for directional motivated reasoning in the context of climate change beliefs has recently been questioned. To illustrate, Druckman and McGrath (2019) propose that individuals may not engage in directional motivated reasoning when assessing climate change information. Instead, individuals may evaluate new evidence aiming to arrive at an accurate conclusion, independent of their prior beliefs. Still, individuals may vary in how they assess the credibility of new information. Thus, observational studies that consider the evidence of bias or political differences as motivated reasoning should be read with some caution.

## Discussion: Future Research and Wider Implications

We have reviewed the state of knowledge on the links between personal experience, prior beliefs and climate change concern/action. Although scholars have been researching the topic for more than a decade, our review shows that the empirical evidence remains mixed. There is some evidence that local weather experiences can generate climate change concern, but the capacity for personal experience to promote action may rely upon the experience first being attributed to climate change (Ogunbode et al., 2019). Attributions may be determined by prior beliefs about climate change, political ideology and/or party identification (Howe and Leiserowitz, 2013). Additionally, the evidence for extreme weather events influencing climate change concern and actions remains limited, with a distinct focus on flooding.

Overall, our review highlights the importance of examining the role of motivational and cognitive biases to help explain how and why weather experiences and prior beliefs may shape individual's climate change perceptions. Behavioral decision research suggests that rare extreme weather events will have limited impact on judgments and decisions, unless they have occurred recently (Konisky et al., 2016). However, even recent events may have limited impact among individuals who hold strong pre-existing beliefs rejecting the reality of climate change (Myers et al., 2013). Existing evidence on the associations between climate change beliefs and perceived weather is consistent with the notion that people interpret and process personal experiences in a biased way that confirms their prior beliefs through a process of motivated reasoning (Howe and Leiserowitz, 2013). However, many of the existing studies report cross-sectional data, making it difficult to determine a causal relationship between personal experience and climate change beliefs. Therefore, as the opportunities for individuals to witness extreme weather events increase, we encourage researchers to utilize a longitudinal and/or experimental design that allow stronger assessments of causality, as studies using such designs are scarce (e.g., Myers et al., 2013; Sobkow et al., 2017). For example, experimental studies could manipulate participants' experience with extreme events in game-like settings (Sobkow et al., 2017) or priming of heat-related cognitions (Joireman et al., 2010).

Differences in methodological approaches across studies should also be highlighted, as the mixed findings outlined may be partly due to such differences. Firstly, some studies use subjective (self-reported) experiences, whereas others examine the influence of objectively measured weather variables on climate change beliefs. Secondly, studies examine a range of independent and dependent variables, which differ in their spatial coverage, time frame, data source and values used. The phrasing of survey questions is also varied, and collectively these differences may in some cases hinder the detection of a relationship between experience and beliefs. Future work could therefore aim to synthesize research designs and methods to aid systematic comparisons, ideally using standardized measures. Finally, most research has been conducted in the U.S. and the U.K. at varying levels (e.g., community, county, state and national) with a distinct focus on local temperature anomalies and flooding. Future research examining different populations and experiences of different forms of extreme weather (e.g., heatwaves and droughts) would help to examine the generalizability and boundary conditions of the findings reviewed.

Gaining a solid understanding of the mechanism underpinning the associations between prior beliefs, personal experience, and concern and action about climate change (or lack thereof) will be critical for the development of effective risk communication strategies suitable for diverse audiences and may also help to inform debiasing interventions. While communicating about climate change risk and the importance of personal action to adapt and mitigate climate change, we suggest communications should also consider the role of non-analytical processes such emotion, the use of imagery and social/group norms to promote climate change efficacy (Hornsey et al., 2021). More work is needed examining what motivates an individual to attribute a personal experience to climate change (e.g., values, worldviews, social structures etc.) as this has been shown to be a key factor in driving mitigation responses (Ogunbode et al., 2019). The role of affective reactions should also be investigated further, as affective reactions to climate change risks may also be subject to moderation by prior attitudes and beliefs (Swim et al., 2009). Yet, intense negative affect can induce fear in some individuals, and as a result lead to avoidant behaviors and denial (Taylor et al., 2014). Finally, future work should examine in more depth the influence of event recency, personal and financial damages, local warming, and psychological and social contexts; all of which may shape how individuals perceive and interact with weather-related experiences.

## Author Contributions

KS conducted the literature search and the literature review. KS, EK, SR, and YO have contributed equally to the finalization of the manuscript. All authors contributed to the article and approved the submitted version.

## Conflict of Interest

The authors declare that the research was conducted in the absence of any commercial or financial relationships that could be construed as a potential conflict of interest.
