# Supplementary material for: The Role of Personal Experience and Prior Beliefs in Shaping Climate Change Perceptions: A Narrative Review
Source: Front Psychol. 2021 Jul 2;12:669911. doi: 10.3389/fpsyg.2021.669911 (PMC8284052; doi:10.3389/fpsyg.2021.669911)
Supplement: Supplementary file 1 [file Table_1.docx]

**Table 1:** Supplementary materials for The Role of Personal Experience and Prior Beliefs in Shaping Climate Change Perceptions: A Narrative Review - Basic characteristics of the studies reviewed and main results.

| **Citation** | **Location** | **Sample size** | **Methodology** | **Variables of interest** | **Summary of results** |
| --- | --- | --- | --- | --- | --- |
| Akerlof et al, 2013 | United States | n = 765 | Observational survey data | Perceived personal experience of global warming (IV)  Perceptions of global warming risk (DV)  Control variables: (1) gender; (2) age; (3) education; (4) income; (5) political affiliation; and (6) cultural worldviews. | In Algar County, Michigan, perceived personal experience of global warming was associated with heightened global warming risk perceptions. |
| Bergquist et al, 2019 | United States | n = 209 | Longitudinal survey data (September, 2017) | Personal experience of extreme weather event (IV)  Climate change beliefs; Intentions to act (DVs)  Control variables: (1) gender; (2) age; (3) environmental concern; (4) political preferences; and (5) subjective income. | Experiencing hurricane Irma was associated with stronger negative emotions toward climate change, increased certainty the hurricane was caused by climate change and increased willingness to pay higher taxes. |
| Brody et al, 2008 | United States | n = 512 | Observational survey data coupled with climate data | Climate change risk perception (DV)  Physical variables: (1) distance to coast; (2) relative elevation; (3) sea level rise; (4) floodplain; (5) temperature trend; (6) economic damage; (7) injuries and fatalities from natural hazards; (8) property damage; (9) fires; (10) state CO_2_ emission and per capita CO_2_  Control variables: (1) ecological values; (2) knowledge; (3) perceived efficacy; (4) network interest; (5) education; (6) income; and (7) gender | Past local temperature changes (i.e. number of warmer-than-average days per year from 1948 to 2005) did not significantly influence climate change risk perceptions. |
| Brooks et al, 2014 | United States | Survey 1 (2004), n = 1093  Survey 2 (2007), n = 832 | Observational survey data coupled with weather data | Deviation from normal temperature (IV)  Degree of concern about global warming and climate change (DV)  Control variables: (1) education; (2) income; (3) political party; (4) political ideology; (5) gender; (6) age; (7) knowledge; and (8) economic concern. | Deviation from mean temperature on the date of the survey was significantly associated with higher levels of climate change concern. |
| Broomell et al, 2017 | United States | n = 207 | Experimental -  two framing conditions labelled weather  (interpreting a temperature as abnormal weather) and climate (interpreting a temperature as evidence of GW). | Beliefs about global warming (IV)  Perceptions of temperature (DV)  Control variables: (1) age; (2) education; (3) knowledge about causes of global warming; and (4) political ideology. | In a randomized experiment, participants were aware when temperatures were abnormally hot, but classify less extreme abnormalities differently based on their beliefs in global warming. |
| Brulle et al, 2012 | United States | n = 84,086 (data from 74 separate surveys conducted from 2002 – 2010) | Observational survey data | Extreme weather events; scientific information; mass media coverage; media advocacy; elite cues (IVs)  Public concern over global climate change (DV)  Control variables: (1) unemployment rate; (2) gross domestic product; (3) war deaths in Iraq and Afghanistan; and (4) price of oil. | Elite cues and structural economic factors had the largest effect on the level of public concern about climate change. Weather extremes have no effect on aggregate public opinion. While media coverage exerts an important influence, this coverage is itself largely a function of elite cues and economic factors. Promulgation of scientific information to the public on climate change has a minimal effect. |
| Carlton et al, 2016 | United States | Survey 1 (2012), n = 7836  Survey 2 (2013), n = 5478 | Observational survey data | Drought severity; drought beliefs (IVs)  Climate change beliefs; risk perceptions; attitudes towards adaptation (DV)  Control variables: (1) age; (2) gender | Neither climate change beliefs nor attitudes toward adaptation changed significantly as a result of the drought. Risk perceptions did change, however, with advisors becoming more concerned about risks from drought and pests and less concerned about risks related to flooding and ponding. Though increased risk perceptions were significantly associated with more favourable adaptation attitudes, the effects were not large enough to cause an overall shift to more favourable attitudes toward adaptation. |
| Carmichael and Brulle, 2017 | United States | n = 84086 | Observational survey data | Availability of scientific information; extreme weather events; political disclosure (IVs)  Climate change threat index (DV)  Intermediary variables: (1) Mass media coverage  Control variables: (1) unemployment rate; (2) gross domestic product. | Weather events had relative small effects on climate change concern. Only extreme drought conditions significantly increased public concern about climate change. |
| Dai et al, 2015 | China | n = 1054 | Observational survey data | Perceived experiences with extreme weather events; physical or financial damages due to extreme weather events (IVs)  Global climate change beliefs (DV)  Control variables: (1) education; (2) gender; (3) age; (4) income; and (5) number of children in the household | Perceived experiences with several extreme weather events (particularly heatwaves) in five Chinese cities were strongly associated with climate change beliefs. Physical or financial damages due to these events lead to even stronger relationships. |
| Demski et al, 2017 | United Kingdom | Respondents who reported direct flooding impacts (n = 162) were compared with a nationally representative sample (n = 975). | Observational survey data | Direct and indirect effects of flood experience (IV)  Mitigation intentions and policy support (DV)  Mediators: (1) negative emotions; (2) climate change concern; and (3) personal issue salience. | Direct experience of flooding lead to greater perceived personal vulnerability and risk perceptions of climate change, and support for mitigation and adaptation policies. |
| Deryugina, 2013 | United States | n = 7847 | Longitudinal survey data (2003-2010) | Short-term temperature fluctuations; long-term temperature fluctuations (IVs)  Global warming beliefs (DV) | Short term temperature fluctuations (1 day – 2 weeks) had no effect on global warming beliefs. However, longer periods of abnormally warm or cold temperatures (1 month – 1 year) did predict global warming beliefs. Only respondents with a conservative political ideology were affected by temperature abnormalities. |
| Egan and Mullin, 2012 | United States | n = 6508 | Observational survey data coupled with weather data | Short-term temperature fluctuations (IV)  Global warming beliefs (DV) | Short term temperature fluctuations that occurred 1 week prior to interview were associated with climate change beliefs. However, the effect decayed quickly and did not induce permanent attitude change. |
| Goebbert et al, 2012 | United States | n = 7968 | Observational survey data coupled with climate data | Local weather change; individual-level characteristics; political ideology and culture (IVs)  Perception of local weather change (DV) | Actual temperature fluctuations were a weak predictor of perceptions of local temperature change, but better predictors of perceived droughts and flooding. Beliefs about changes in local temperature were more heavily politicized than beliefs about local precipitation patterns. |
| Hamilton et al, 2015 | United States | n = > 28000 | Observational survey data | Predictors: (1) age; (2) gender; (3) education; and (4) political party.  Climate change beliefs (DV) | Political identity significantly predicts individual’s climate change beliefs. Acceptance of climate change rises with education among Democrats and Independents, but not so among Republicans. |
| Hamilton and Stampone, 2013 | New Hampshire, United States | n = 5000 | Observational survey data coupled with weather data | Temperature anomalies; age; gender; education; political identity; self-assessed understanding (IVs)  Climate change beliefs (DV) | Climate change beliefs were predicted by temperature anomalies on the interview day and the previous day. Temperature effects were concentrated among those who identified as political independents. |
| Hart and Nisbet, 2012 | New York, United States | n = 240 | Experimental – two stimulus conditions exposing participants to news stories about possible climate change health impacts on different groups (vs. control condition) | Climate change beliefs (IV)  Support for government action on climate mitigation (DV)  Moderator: Political partisanship  Mediator: Social identification with potential victims  Control variables: (1) age; (2) gender; and (3) level of education | Embedded social identity cues interacted with political orientations to amplify public polarization on climate change. Knowledge and beliefs about climate change were not associated with support for climate mitigation policies. |
| Howe, 2018 | Norway | n = 9669 | Observational survey data coupled with seasonal climate data | Climate change beliefs; spatial climate data (IV)  Predictors: (1) age; (2) gender; (3) education; and (4) political orientation  Perceptions of local weather (DV) | Respondent’s perceptions were sensitive to observed differences in both temperature and precipitation. Climate change beliefs had a significant effect on perceptions of seasonal temperature, but smaller effects on perceptions of seasonal precipitation. |
| Howe and Leiserowitz, 2013 | United States | n = 1010 | Observational survey data coupled with climate data | Predictors: (1) climate change beliefs; (2) age; (3) education; (4) ethnicity; and (5) political party  Perceived personal experience of local weather anomalies (DV) | Subjective experiences of seasonal average temperature and precipitation during the previous winter and summer were related to recorded weather conditions and beliefs about global warming. |
| Joireman et al, 2010 | North-western United States | Study 1, n=93  Study 2, n = 42  Study 3, n = 159 | Study 1: Observational survey data  Study 2: Experimental - priming of words related to heat (vs. control condition)  Study 3: Observational survey data | Outdoor temperature; anchoring regarding the amount of temperature increase  expected in the future (IVs)  Belief in global warming; willingness to pay to reduce global warming (DV)  Control variables: (1) environmental values; (2) political party | Study 1: Significant positive correlation between the outdoor temperature and beliefs in global warming.  Study 2: People were more likely to believe in global warming when they had first  been primed with heat-related cognitions.  Study 3: People were more likely to believe  in global warming and more willing to pay to reduce global warming when they had first been exposed to a high vs. a low anchor for future increases in temperature. |
| Konisky et al, 2016 | United States | n = >130000 | Observational survey data coupled with extreme weather events data | Extreme weather episodes (IV)  Climate change beliefs (DV)  Control variables: (1) age; (2) gender; (3) minority; (4) marital status; (5) income; (6) education; and (7) church attendance | There was a positive relationship between experience of extreme weather activity and expressions of concern about climate change. However, the effect of extreme weather on public concern was only significant for recent weather events. |
| Krosnick et al, 2006 | United States | Study 1 (n = 1413)  Study 2 (n = 758). | Observational survey data | Perceived personal experience of global warming (IV)  Climate change beliefs (DV) | Respondents who believed they had witnessed rising temperatures in recent years were more likely to believe in the existence of global warming. |
| Leiserowitz, 2006 | United States | n = 673 | Observational survey data | Holistic affect and affective imagery; values; sociodemographics (IVs)  Global warming risk perception; climate change policy preferences; climate tax policy preferences (DVs)  Control variables: (1) gender; (2) age; (3) income; (4) educational attainment; (5) race or ethnicity; (6) main source of news; (7) party identification; (8) political ideology; and (9) voter registration | Risk perceptions and policy support were strongly influenced by experiential factors, including affect, imagery, and values. Public responses to climate change were influenced by both psychological and socio-cultural factors. |
| Li et al, 2011 | United States & Australia | Study 1 (n = 582)  Study 2 (n = 251) | Observational survey data | Perceived local weather change (IV)  Global warming concern and beliefs (DV) | Respondents who thought the day they were surveyed was warmer than usual believed more in and had greater concern about global warming than did respondents who thought the day was colder than usual. Respondents also donated more money to a global-warming charity if they thought the day seemed warmer than usual. |
| Lujala et al, 2015 | Norway | n = 1334 | Observational survey data | Direct personal experience of climate-related hazard (IV)  Climate change perceptions (DV)  Control variables: (1) age; (2) gender; (3) education; (4) income; and (5) political orientation | Respondents who reported direct personal experience of damage due to a natural-hazard event were more likely to be concerned about the personal consequences of climate change. However, this direct experience did not affect their likelihood of listing climate change as a major threat for the future. |
| Marlon et al, 2019 | Florida, United States | n = 1046 | Observational survey data coupled with weather data | Predictors: (1) subjective local weather change; (2) subjective hurricane experience; (3) climate change beliefs; (4) gender; (5) political ideology; and (6) outdoor professions.  Climate change risk perceptions (DV) | Floridians were unable to detect five-year increases in temperature but could detect changes in precipitation. Climate change risk perceptions were more strongly predicted by subjective experiences of environmental change, personal beliefs about climate change, and political ideology, compared to local weather variables. |
| McCright et al, 2014 | United States | n = 1020 | Observational survey data coupled with weather data | Predictors: (1) political ideology; (2) party identification; (3) education; (4) gender; and (5) age.  Mediators: (1) perceived scientific agreement; and (2) global warming beliefs.  Perceived local winter warming; Global warming as the main cause (DVs) | Temperature anomalies influence perceived warming but not attribution of such warmer-than-usual winter temperatures to global warming. Rather, the latter is influenced more by perceived scientific agreement; beliefs about the current onset, human cause, threat and seriousness of global warming; and political orientation. |
| Myers et al, 2013 | United States | n = 3200 | Longitudinal survey data (2008 & 2011) | Personal experience  Belief certainty  Control variables: (1) gender; (2) education; (3) income; and (4) political ideology | Perceived personal experience of global warming led to increased belief certainty. At the same time, high belief certainty influenced perceptions of personal experience. |
| Ngo et al, 2020 | Vietnam | n =1086 | Observational survey data | Individual knowledge; Flood experience; Community participation (IVs)  Perceived vulnerability; Perceived severity; Perceived adaptive capacity; Intention to take actions for reducing flood risks (DVs)  Control variables: (1) age; (2) gender; (3) education; (4) income; and (5) poverty status | Significant association of flood and climate change risk perceptions with individual’s flood experience, climate change knowledge, frequency of community participation and socio-demographic factors. Flood experience was the most influential driver of flood-related risk perceptions but weak for climate change-related risk perceptions and behavioural intentions. |
| Ogunbode et al, 2019 | United Kingdom | n = 1997 | Observational survey data | Predictors: (1) flooding experience; (2) climate change beliefs; (3) political affiliation; and (4) perceived normative cues  Moderator: subjective attribution  Perceived threat from climate change; mitigation intentions or policy support (DVs) | Personal experience of a flooding event directly predicted perceived threat from climate change, and indirectly predicted climate change mitigation responses, among individuals who subjectively attributed the floods to climate change. |
| Palm et al, 2017 | United States | n = 9500 | Longitudinal survey data (2010-2014) | Predictors: (1) experience with weather anomalies; (2) party identification; (3) education; (4) age; (5) gender; and (6) ethnicity  Climate change opinion (DV) | Democrats strongly believed that climate change is occurring, and that immediate action is required. Republicans, on the other hand, generally remained convinced that climate change is not occurring or that its seriousness is exaggerated. Direct experience with warmer weather, droughts, and weather-related natural disasters had little effect on climate change opinions. |
| Reser et al, 2011 | Australia and United Kingdom | n = 3096 (Australian sample)  n = 1822 (UK sample) | Observational survey data | Direct experience (IV)  Climate change concern & beliefs; risk perception; willingness to act; climate change distress; psychological adaptation to climate change; previous exposure with natural disasters; self-efficacy (DVs) | Prior direct experience with extreme weather events and natural disasters for Australian respondents showed consistent but modest positive relationships with climate change-related psychological variables such as belief, concern, psychological adaptation, psychological distress, and behavioural engagement. |
| Shao, 2016 | United States | n = 1052 | Observational survey data coupled with weather and climate data | Predictors: (1) age; (2) gender; (3) income; (4) education; (5) religion; (6) ethnicity; (7) political predisposition; (8) local weather; and (9) extreme weather events.  Local weather change; risk perceptions of global warming impacts; global warming priority (DVs) | Pre-existing beliefs about global warming played a dominant role in determining perceptions of weather. Respondents who believed global warming is causing an immediate impact, is serious, and needs to be a priority, motivated by their pre-existing beliefs, were much more likely to perceive the ‘evidence’ of global warming. |
| Shao, 2017 | United States | n = 5916 | Observational survey data coupled with weather and climate data | Political predisposition; religion; attentiveness to news sources; weather & climate (IVs)  Belief in global warming existence; perception of global warming cause; evaluation of global warming impact (DVs)  Control variables: (1) age; (2) race; (3) family income; (4) education; and (5) gender | Warming winters coupled with cooling springs over the past 10 years had positive effects on belief in the existence of global warming.  Republicans and political conservatives were less likely than Democrats and political liberals to perceive temperature rise, to attribute temperature rise to human cause, and to view global warming negatively. |
| Shao et al, 2016 | United States | n = 2001 | Observational survey data coupled with weather and climate data | Local weather and climate; attitudes towards scientists and science; political predispositions; information environment; religious beliefs and attachments; race; gender; income; and age (IVs)  Perceptions about global warming (DV) | Individuals who have experienced long-term warming of summer temperatures coupled with long-term cooling of spring temperatures were significantly more likely to perceive that global warming exists and is due to human activity. |
| Shao and Goidel, 2016 | Gulf Coast, United States | n = 3856 | Observational survey data coupled with weather and climate data | Age; education; income; race and ethnicity; gender; political predisposition; attentiveness to information about climate change; and objective environmental conditions (IVs)  Perceptions of weather conditions; Perceptions of climate change (DVs) | Political orientations rather than local conditions drove perceptions of local weather conditions. Republicans were less likely than Democrats to perceive increasing air temperatures, hurricanes,  droughts, and flooding. Local weather perceptions were found to influence climate change attitudes. |
| Shao et al, 2014 | United States | n = varies from 287 - 1604 | Observational survey data coupled with climate data | Age; education; income; gender; race; religious services attendance; political orientation; weather and climate trends (IVs)  Perceptions of global warming (DV) | Local climate conditions represented by summer temperature trends over a 10-yr time period had a discernible effects on attitudes relating to the immediacy and severity of global warming. |
| Sisco et al, 2017 | United States | 10748 weather events (2011-2014)  Attention measured using ~1.7 million Twitter messages | Social media data coupled with extreme weather events data and daily weather records | Extreme weather experience (type, financial damage, deaths and abnormality); absolute vs. relative levels of weather variables (temperature, wind speed and precipitation) (IV)  Attention to climate change (DV) | The effects of extreme weather experiences were larger directly after weather events occurred compared to directly before. Financial damage associated with the weather events had a positive and significant effect on attention, although the effect was small. |
| Spence et al, 2011 | United Kingdom | n = 1822 | Observational survey data | Flooding experience (IV)  Perceptions of climate change; behavioural intentions (DV)  Control variables: (1) age; (2) gender; and (3) social grade | Those who reported direct flooding experience expressed more concern over climate change. Increased concern about climate change was associated with greater willingness to save energy to mitigate climate change. |
| Taylor et al, 2014 | United Kingdom | n = 1848 | Observational survey data | Beliefs regarding changes in weather; Personal experience of specific weather-related hazards (IVs)  Climate change beliefs (DV)  Control variables: (1) environmental values; (2) age; (3) gender; and (4) education | Heatwaves and hot summers were perceived to have become less common during respondents lifetimes, while flooding, periods of heavy rainfall, coastal erosions and mild winters were perceived to have increased in frequency. Climate change beliefs were positively associated with hot and wet-weather-related events. |
| Whitmarsh, 2008 | South of England, United Kingdom | Semi structured interviews (n = 24)  Survey (n = 589) | Observational survey data | Air pollution affected health; experience of flooding in last 5 years; gender; age; annual income; education; political party; newspaper regularly read; and member of environmental organisation (IVs)  Perceived threat from climate change; climate change considered personally very important; belief that anthropogenic climate change is real; action taken out of concern for climate change (DVs) | Direct experience of flooding had very little effect on respondents understanding of and responses to climate change. Experience of air pollution significantly affected perceptions of and behavioural responses to climate change. |
| Zanocco et al, 2018 | Laurel County, Kentucky, and Winston County, Mississippi; Yavapai County, Arizona, and Lake County, California, United States | n = 489 | Observational survey data | Event proximity; reported personal harm; gender; education; income; age; political ideology and race/ethnicity (IVs)  Climate change views after extreme weather event (DV) | Reported personal and community harm aligned with event proximity and larger community damages. Climate change views were strongly predicted by political ideology. |
| Zaval et al, 2014 | United States | n = 686 | Observational survey data | Perceived temperature deviation (IV)  Belief in and concern for global warming (DV)  Control variables: (1) actual temperature; (2) actual deviation; (3) gender; (4) education; (5) age; (6) income; (7) political affiliation; (8) environmental attitude; and (9) subjective knowledge of the phenomenon | Present temperature anomalies were associated with an overestimation of the frequency of similar past events, which was related to an increased belief in and concern for global warming. |
